# The influence of viral RNA secondary structure on interactions with innate host cell defences

**DOI:** 10.1093/nar/gkt1291

**Published:** 2013-12-13

**Authors:** Jeroen Witteveldt, Richard Blundell, Joris J. Maarleveld, Nora McFadden, David J. Evans, Peter Simmonds

**Affiliations:** ^1^Infection and Immunity Division, Roslin Institute, University of Edinburgh, Easter Bush, Edinburgh, EH25 9RG and ^2^School of Life Sciences, University of Warwick, Coventry, CV4 7AL, UK

## Abstract

RNA viruses infecting vertebrates differ fundamentally in their ability to establish persistent infections with markedly different patterns of transmission, disease mechanisms and evolutionary relationships with their hosts. Although interactions with host innate and adaptive responses are complex and persistence mechanisms likely multi-factorial, we previously observed associations between bioinformatically predicted RNA secondary formation in genomes of positive-stranded RNA viruses with their *in vivo* fitness and persistence. To analyse this interactions functionally, we transfected fibroblasts with non-replicating, non-translated RNA transcripts from RNA viral genomes with differing degrees of genome-scale ordered RNA structure (GORS). Single-stranded RNA transcripts induced interferon-β mediated though RIG-I and PKR activation, the latter associated with rapid induction of antiviral stress granules. A striking inverse correlation was observed between induction of both cellular responses with transcript RNA structure formation that was independent of both nucleotide composition and sequence length. The consistent inability of cells to recognize RNA transcripts possessing GORS extended to downstream differences from unstructured transcripts in expression of TNF-α, other interferon-stimulated genes and induction of apoptosis. This functional association provides novel insights into interactions between virus and host early after infection and provides evidence for a novel mechanism for evading intrinsic and innate immune responses.

## INTRODUCTION

Recognition of invading microorganisms and coordinating a defence response are critical steps for host organisms in their perpetual battle with pathogens. Viruses are generally detected initially by host sensors for foreign nucleic acids, including double-stranded RNA duplexes or viral DNA in the cytoplasm and single- or double-stranded RNA or DNA in endosomes (1,2). Reflecting a long-term, evolutionary arms race between viruses and their hosts, viruses have evolved an extensive and highly diverse range of counter-measures that block their recognition or impair the action of interferon and other effector mechanisms (3).

With their relatively small genome size, RNA viruses, in particular, have become very efficient at making the best use of their seemingly simple genomes beyond merely coding for structural and non-structural proteins required for virion assembly and genome replication. Many RNA viral genomes contain additional genes to inhibit or divert innate cell defences, typically embedded within other viral genes in alternative reading frames (4), such as the PB1-F2 protein of influenza A virus (5) or VF1 of murine norovirus (MNV) (6). Single-stranded RNA genomes or transcripts from DNA viruses additionally may internally base-pair and form RNA secondary or tertiary structures that interact with host cell defences. These include the EBER and VA transcripts of Epstein–Barr virus and adenovirus that interact with and inhibit PKR (7,8) and a sub-genomic highly structured RNA sequence derived from the 3′-end of flavivirus genomes that is required to induce cytopathology and pathogenicity in mice (9). The presence of structured RNA can also influence the effectiveness of RNA interference and consequently the host innate immune response (10–12).

RNA viruses additionally and frequently possess replication and translation initiation elements that depend on internal RNA base pairing for their interactions with viral and cellular protein and components of the ribosome. Amongst positive-stranded RNA viruses, picornaviruses and two genera in the *Flaviviridae* use extensively base-paired internal ribosomal entry sites (IRESs) that recruit ribosomes and initiate translation (13–16). RNA secondary structure elements also regulate transcription, and several steps of the virus replicative cycle. These include *cis*-acting replication element (CREs) in picornaviruses (17–20) implicated in VPg uridylylation and long-range RNA–RNA kissing-loop interactions that form a key component in the encapsidation process of several retroviruses (21,22)

RNA structures characterized to date are discrete elements with defined functions (where characterized) and specific phenotypic effects. However, using bioinformatic and biophysical methods we have demonstrated that many genera and families of positive-sense, single-stranded animal and plant RNA viruses exhibit extensive regions of RNA structure extending throughout the genome (23,24). We have designated this type of structure genome-scale ordered RNA structure (GORS) to make clear its whole-genome distribution which contrasts with the diverse range of discrete replication and cell interaction elements possessed by most RNA viruses. Indeed, GORS is found in only a subset of viruses and its presence is not conserved between genera of virus families, ruling out that the possibility that it is involved in any conserved aspects of the viral life cycle (23,24). Instead, the presence of GORS correlated with the ability of the virus to persist in its host. Persistence of a virus in its host suggests it is able to defeat or circumvent both the innate and subsequent acquired immune responses. We therefore speculated that this correlation between GORS and persistence indicates a possible role of extensive secondary structures in the evasion of the innate immune response, either by preventing primary recognition or obstructing the downstream signalling cascade.

The detection of viral infection by hosts relies on sensors termed pattern-recognition receptor (PRR) molecules that detect specific pathogen-associated molecular patterns (PAMP) (25). As we have shown that there is a profound difference in the level of folding of viral genomes, PRRs that recognize non-self nucleic acids are likely to be the most relevant in the case of GORS. Conventionally, these include the endosomal Toll-like receptors (TLRs) TLR3, TLR7/8 and TLR9, the cytoplasmic sensors RIG-I, MDA-5 and LGP2 (2,26–28) and components of the effector end of the IFN response, protein kinase R (PKR) and RNAseL (26,29,30). Each of these sensors specializes in detection of a specific type of ribonucleic acid and, upon activation, initiate complex-signalling cascades. These lead to the production of type-I interferons and pro-inflammatory cytokines that stimulate antiviral responses in the infected cells and those surrounding it to prevent virus replication.

We investigated the possibility that GORS modulates the recognition of viral RNA in a manner that contributes to viral persistence. Genomic RNA sequences from a range of persistent and non-persistent viruses that differed in their degree of RNA folding were transfected into cells and cellular responses recorded. This included downstream induction of IFN-β, the formation of antiviral stress granules (SGs) (31,32), activation of other genes associated with innate cellular responses such as IL8 and downstream apoptosis. Specific inhibitors and RNAi-mediated inhibition of different components in signalling pathways were used to identify at what stage differential recognition of structured and unstructured RNAs occurred.

## MATERIALS AND METHODS

### Cell culture

A549, Huh7, Huh7.5, RD and NIH3T3 cells were propagated in Dulbecco’s modified Eagle medium supplemented with 10% foetal calf serum, penicillin (100 U/ml) and streptomycin (100 μg/ml) and maintained at 37°C and 5% CO_2_. The cell lines A549/pr(IFN-β).GFP, A549/pr(IFN-β).GFP-NS3/4a, A549/pr(IFN-β).GFP-Npro, A549/pr(IFN-β).GFP-PIV5-V and the A549 cells overexpressing shRNAs targeting RIG-I, MDA-5 and PKR were kindly provided by Professor R. Randall, University of St. Andrews (33,34). Cell proliferation was measured using WST-1 reagent (Roche) according to the manufacturer’s instructions. For assessing the role of PKR, cells were pre-treated with 2-aminopurine (Sigma-Aldrich) or solvent control [PBS:glacial acetic acid (200:1)] and was kept in the medium during incubation. The imidazolo-oxidole inhibitor C16 (Sigma-Aldrich) was also used to pre-treat cells at 1 μM or DMSO as control and kept in the medium during incubation. Chloroquine (Sigma-Aldrich) dissolved in PBS was used at 10 μM as pre-treatment and during the incubation period.

### Viral and cellular RNA

For the production of RNA for transfection, template was amplified from viral cDNA clones or viral RNA by PCR using a forward primer that contained a T7 promoter sequence and an antisense primer at ∼4 KB distance from the sense primer binding site (Supplementary Table S1). After gel purification and subsequent phenol/chloroform extraction of amplified DNA, RNA was transcribed using T7 *in vitro* transcription with the PCR fragment as template (Megascript, Ambion). The integrity of transcripts was verified by gel electrophoresis and on a 2100 Agilent Bioanalyser before transfection. RNA was purified by lithium chloride precipitation, washed with 70% ethanol and re-suspended in nuclease-free water. Cellular RNA was extracted from A549 cells using RNeasy (Qiagen) spin columns according to the manufacturer’s instructions including DNase treatment.

For immunofluorescence detection, RNA was *in vitro* transcribed and purified as described above, but with 1:4 Biotin-11-UTP (Ambion): UTP in the transcription mixture. RNA was dephosphorylated using FastAP thermosensitive alkaline phosphatase (Fermentas) as described before (35) and purified by lithium chloride precipitation. For gel purification, RNA in excised fragments was isolated using the Zymoclear Gel RNA recovery kit (Zymo Research).

### Transfections

Approximately 1.0 × 10^5^ cells were seeded a day before transfections into 24 well plates. RNA was transfected using Lipofectamine 2000 (Invitrogen); RNA in 50 μl of DMEM was incubated for 5 min with 1 μl of Lipofectamine 2000, incubated for 20 min and added to wells containing target cells at 90% confluency.

### Quantitative real time polymerase chain reaction (qRT-PCR)

Transfected cells were incubated for 8 h and RNA was extracted and isolated using the RNeasy (Qiagen) or Illustra RNAspin kit (GE Healthcare) according to the manufacturer’s instructions, both including DNase treatment. IFN-β mRNA levels were measured using qRT-PCR using primers listed in Supplementary Table S2 and calculated as fold induction over mock transfected cells. As a result, IFN-β induction levels comparable or even exceeding that of the synthetic dsRNA analogue poly I:C were observed. For each sample ∼1 μg of RNA extracted from the cells was reverse transcribed using M-MLV (Promega) and random hexamers according to manufacturer’s instructions. The cDNA was amplified using either a Step-One-Plus thermocycler (Applied Biosystems) or Rotorgene (Qiagen) using Sensifast Sybr green mastermix (Bioline). The real-time qRT-PCR primers were either published before or designed using qPrimerDepot (http://primerdepot.nci.nih.gov/) and are listed in Supplementary Table S2.

### RNA secondary structure prediction

Secondary structure predictions were performed using a locally implemented compiled version of the program UNAFold (36) accessed from http://mfold.rna.albany.edu/?q=DINAMelt/software. For calculation of mean folding energy differences (MFEDs) between native RNA-viral sequences and sequence order-randomized controls, 50 copies of each sequence was generated using the algorithm NDR that preserves dinucleotide frequencies of the native sequence, as implemented in the program Folding Energy Scan in the SSE package (23). Minimum free energies (MFEs) of each native sequence were compared with the mean MFE of the NDR scrambled controls. The arithmetical difference reflects the contribution of sequence order to RNA structure, expressed as an MFED value. For sequences longer than 500 bases, MFEDs were calculated by averaging values of 300 base fragments incrementing by 150 bases across the sequence.

### Generation of synthetic MNV and hepatitis C virus transcripts

From non-coding region (Region 1; positions 1 1147–2467) of the pT7:MNV3 clone (37), 1.3-Kb transcripts were synthesized. Sequences between these positions were permuted by the program Sequence Scramble in SSE, using the CDLR algorithm (38). This permuted nucleotide sequence order while retaining coding order and base composition (including dinucleotide frequencies) of the native sequence. MFED values of 50 000 permuted sequences approximated to a normal distribution ranging from −3.6% to 14.5%. From this distribution, an unstructured (US) sequence with an MFED of ∼0 (−0.4%) and a re-stabilized (RS) second sequence with an MFED of 9.0%, corresponding to that of the native sequence, were selected and synthesized (Life Sciences, Paisley, UK). Transcripts were additionally made from native (WT) and US sequences derived from the non-structural region of the Con1 clone (AJ238799) of hepatitis C virus (HCV) between positions 6529–7635 [as numbered in the H77 reference genome (39)]. The US mutant had an MFED value of −1.3%, compared to the WT sequence of 10.9%.

### Immunofluorescence

For immunofluorescence analysis, cells were grown on cover slips in 24-well plates. After treatment, the cells were washed with PBS and fixed with 4% formaldehyde for 30 min at room temperature. After washing in PBS, the cells were blocked and permeabilized with PBS supplemented with 10% foetal calf serum and 0.1% Triton X-100. Subsequent incubation with the primary and secondary antibodies was performed in the same blocking/permeabilization buffer for 1 h at room temperature. Primary antibodies directed against Ras-GAP SH3 domain-binding protein (G3BP) were obtained from BD Pharmingen. Secondary antibodies were anti-rabbit Alexa Fluor 488 (Life Technologies), anti-mouse Alexa Fluor 594 (Life technologies) and anti-biotin CF633 (Sigma).

### PKR Detection

Phosphorylated PKR was detected by Western-blot analysis of cell lysates collected at different time points post-transfection. Cells were lysed in 2% sodium dodecyl sulphate (SDS) in 62.5 mM Tris–HCl, pH 6.8, 5% 2-mercaptoethanol and electrophoresed on a 10% denaturing SDS-polyacrylamide gel followed by immunoblotting. Phosphorylated PKR was detected by specific antibody [anti-phospho-PKR (Thr451), Merck-Millipore] followed by an HRP-conjugated antibody (Thermo scientific) and ECL detection.

## RESULTS

### Recognition of viral RNA

Differential recognition of viral genomic RNA with or without large-scale RNA secondary structure is most likely manifested early after virus entry and before establishment of replication complexes and consequent sequestration of dsRNA replication intermediates in the endoplasmic reticulum. To functionally characterize this initial recognition of viral RNAs, RNA sequences of defined sizes were transfected into A549 cells and expression of IFN-β mRNA monitored by quantitative RT-PCR. Transcripts of ∼4 kb were chosen from a range of representative RNA viruses, avoiding known defined RNA structures (such as CRE-associated stem-loops involved in replication functions) and IRES elements. In addition, transcripts were not capped and could not therefore be translated and their integrity was verified before transfection by gel electrophoresis and using a 2100 Agilent bioanalyser. Transfected cells were incubated for 8 h and RNA was extracted. IFN-β mRNA levels were measured using qRT-PCR and calculated as fold induction over mock transfected cells.

**Figure 1.**
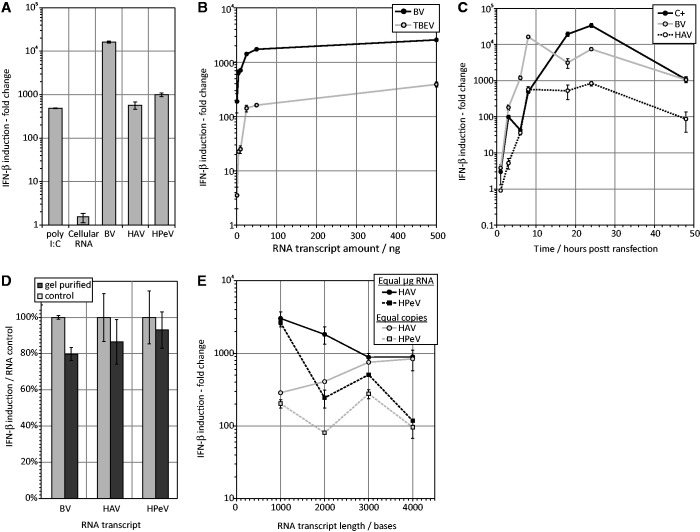
IFN-β induction by transfected viral RNA. (**A**) Induction of IFN-β in A549 (human fibroblast) cells at 8 h following transfection of poly-I:C, human cellular RNA and viral RNA transcripts. The *y*-axis records IFN-β levels (log scale) as a ratio of the mock transfected (no RNA) control (fold-induction). (**B**) Dose response of IFN-β to two representative transcripts. (**C**) Time course of IFN-β induction post-transfection. (**D**) Comparison of IFN-β induction between mock-purified (control) *in vitro* RNA transcripts and after gel purification (**E**) Induction of IFN-β by transcripts of HAV and HPeV of different lengths. Amounts transfected were normalized to equal RNA copies or equal mass of RNA transfected (legend). In all experiments, bars or graph points show mean values of three replicates; error bars show ± 1 SD around mean value.

Transfections were initially performed using 5 ng of RNA per 200,000 cells (ie. per well of a 24-well plate) derived from viruses that showed low MFED values and that were associated with non-persistent infections (Tables 1 and 2). These included HAV, HPeV, TBEV and bunyanwera virus (large segment; BV). Transcripts from BV, HAV and HPeV induced high levels of IFN-β mRNA 8 h post-transfection, similar to that of the poly-I:C positive control (Figure 1A), while transfection of equivalent amounts of human cellular RNA invoked no response (lane 2). Induction was dose-dependent, increasing between up to 2571-fold (BV) and 389-fold (TBEV) with 1–500 ng input RNA, with relatively little increase with amounts of RNA >50 ng (Figure 1B). IFN-β induction occurred maximally around 8 h post transfection for viral RNA sequences and ∼24 h post transfection for the poly-I:C control (Figure 1C).

**Table 1.** Sources of RNA transcripts used for transfection

Virus	Name	Family	Genus	Accession number
BV	Bunyamwera virus	*Bunyaviridae*	*Orthobunyavirus*	X14383
CaCV	Canine calicivirus	*Caliciviridae*	*Calicivirus*	AB070225
HAV	Hepatitis A virus	*Picornaviridae*	*Hepatovirus*	M59808
HCV	Hepatitis C virus	*Flaviviridae*	*Hepacivirus*	AB047639
HPeV 1	Human parechovirus type 1	*Picornaviridae*	*Picornavirus*	L02971
HPgV	Human pegivirus	*Flaviviridae*	*Unclassified*	AF121950
MNV1	Murine norovirus 1	*Caliciviridae*	*Norovirus*	DQ285629
MNV3	Murine norovirus 3	*Caliciviridae*	*Norovirus*	JQ658375
MeV	Measles virus	*Paramyxoviridae*	*Morbillivirus*	AY486083
PEMV-2	Pea enation mosaic virus-2	*Umbravirus*	*Umbravirus*	AY714213
PLRV	Potato leafroll virus	*Luteoviridae*	*Polerovirus*	AY138970
PV	Poliovirus	*Picornaviridae*	*Enterovirus*	V01149
RV	Rubella virus	*Togaviridae*	*Rubivirus*	M15240
SFV	Semliki forest virus	*Togaviridae*	*Alphavirus*	X04129
SeV	Sendai virus	*Paramyxoviridae*	*Respirovirus*	M30202
SiV	Sindbis virus	*Togaviridae*	*Alphaviridae*	J02363
TBEV	Tick borne encephalitis virus	*Flaviviridae*	*Flavivirus*	U39292
TMEV	Theiler’s murine encephalitis virus	*Picornaviridae*	*Theilovirus*	X56019

**Table 2.** RNA-folding energies and composition of RNA transcripts

Transcript	MFE^a^	MFED (%)^b^	GORS^c^	G + C	CpG^d^	UpA^e^
CaCV	−77.0	16.0	Yes	0.468	0.766	0.505
PLRV	−84.2	13.6	Yes	0.508	0.809	0.652
HPgV	−115.1	12.0	Yes	0.594	0.692	0.575
TMEV	−73.7	6.5	Yes	0.502	0.586	0.590
PEMV	−93.7	6.5	Yes	0.559	0.735	0.758
MNV3	−99.1	6.3	Yes	0.579	0.605	0.455
SFV	−84.4	5.9	Yes	0.526	0.853	0.816
HCV	−101.9	5.5	Yes	0.588	0.686	0.802
MNV1	−96.2	4.0	Yes	0.572	0.605	0.500
TBE	−93.8	2.8	No	0.549	0.527	0.369
MeV	−70.2	1.3	No	0.534	0.581	0.601
RV	−118.2	1.0	No	0.716	1.130	0.678
SeV	−72.0	0.8	No	0.47	0.517	0.789
PV	−70.3	0.7	No	0.475	0.568	0.855
HPeV	−62.2	0.5	No	0.392	0.225	0.678
SiV	−71.6	0.1	No	0.513	0.863	0.782
HAV	−61.7	−2.1	No	0.366	0.069	0.564
BV	−51.2	−3.6	No	0.335	0.258	0.802

^a^Minimum folding energy (Kcal/mol).

^b^MFE difference from sequence order randomized control.

^c^Threshold of 3% MFED value.

^d^Observed to expected value: 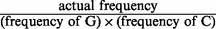.

^e^Observed to expected value: 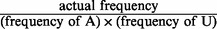.

To check for contamination of the RNA preparations with partial or reverse transcripts, RNA fragments of the expected size were excised from a gel, purified and their integrity confirmed on an Agilent bioanalyser. Their ability to induce IFN-β expression was quantified in parallel with unpurified transcripts; IFN-β levels were similar between directly transcribed RNA and the same RNA sequences after stringent gel purification (Figure 1D). To investigate the influence of sequence length on IFN-β induction PCR-generated DNA templates of 1–4 kb from HAV and HPeV were generated (primers listed in Supplementary Table S1) and transcribed *in vitro.* These were transfected into A549 cells with RNA amounts equal to those used for the 4-kb fragments (10 ng) or amounts equivalent in copy number of those used for the 4-kb fragments. Although IFN-β levels showed some variability between the two viral sequences, IFN-β induction was comparable between different fragment lengths when equal RNA copies were transfected (grey filled symbols; Figure 1E). Maintaining the same mass of RNA resulted in a decrease in IFN-β induction with an increasing size (and decrease in RNA molecules) (black filled symbols).

**Figure 2.**
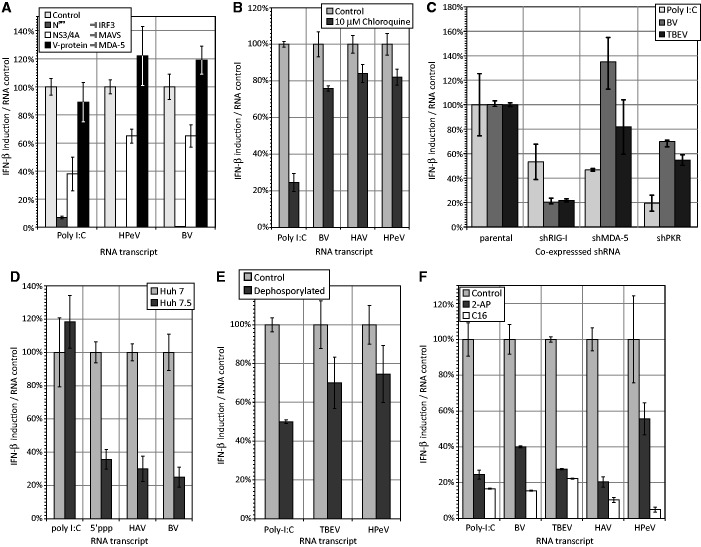
Recognition and signalling pathways following RNA transfection. (**A**) Effect of co-expression of BVDV N^Pro^ (inhibits IRF3), HCV NS3/4A (inhibits MAVS/IPS-1/CARDIF) and V-protein (inhibits MDA-5) on IFN-β induction. (**B**) Effect of 10 µM chloroquine (inhibits endosomal acidification and blocks TLR function) on IFN-β induction. (**C**) shRNA knockdown of RIG-I, MDA-5 and PKR and effect on IFN-β induction. (**D**) Transfection of RNAs into Huh7 and HuH7.5 (non-functional RIG-I) hepatoma cells. The 5′ppp is a 5′-phosphorylated dsRNA control for RIG-I. (**E**) Effect of transcript dephosphorylation on IFN-β induction. (**F**) PKR inhibitors 2-amino purine (2-AP) and C16 inhibit IFN-β induction; poly-I:C was used as a control. All graphs were plotted as the mean change in expression from untreated or WT cells; bars represent the mean of three biological replicates and error bars represent ± 1 SD around the mean.

To investigate the components of the IFN-β signalling pathway and PRRs involved in recognition of transfected viral RNA, IFN-β mRNA induction was compared in a range of cell types in which these determinants were either absent or suppressed with specific siRNAs, protein and chemical inhibitors (Figure 2). Results were expressed throughout as IFN-β levels relative to that in untreated or control cell lines. Inhibition of interferon regulatory factor 3 (IRF3)-mediated signalling by BVDV N^Pro^ (40) eliminated IFN-β mRNA induction by viral RNA transcripts and significantly reduced poly-I:C-induced IFN-β RNA levels (Figure 2A). Since BVDV N^Pro^ works by ubiquitinating and degrading IRF3, the abolition of an IFN-β response provides evidence against signalling through other pathways, such as those activated by TLR7 and TLR9 that recruit IRF7. To investigate the role of endosomal TLR3 in recognition, A549 cells were pre-treated with chloroquine, a lysosomotropic agent that prevents endosomal acidification and consequently TLR3 signalling (41). Chloroquine treatment led to minimal reductions in IFN-β expression on transfection of BV, HAV and HPeV RNA sequences compared to untreated cells (Figure 2B), an observation that contrasted with the 75% reduction observed for the partially dsRNA poly-I:C control.

The roles of RIG-I-like receptors (RLRs) in RNA recognition were assessed using two approaches. In the first, an A549 cell line overexpressing the HCV protease (NS3/4A) that inhibits MAVS-dependent signalling by RIG-I and MDA-5, was used. The effect of transfecting HPeV and BV RNA sequences on IFN-B responses was assayed in parallel in an A549 cell line that overexpressed the PIV-5 V protein which specifically inhibits MDA-5 (42) (Figure 2A). Expression of the NS3/4A protein resulted in a marked reduction in IFN-β expression compared to the parental line, whereas expression of the V-protein had no effect. For the second approach, A549 cell lines overexpressing RIG-I and MDA-5 specific shRNAs were generated; RIG-I and MDA-5 mRNA levels were >10% of those expressed in the parental A549 cells (data not shown). Consistent with the previous results, RIG-I specific shRNA expression led to profound reductions in IFN-β mRNA induction compared to control cells while small increases were detected in cells expressing shRNAs against MDA-5 (Figure 2C). Involvement of RIG-I in RNA detection and signalling was consistent with the observation of reduced IFN-β induction in Huh7.5 cells (in which RIG-I is defective) compared to the parental Huh7 cell line (Figure 2D). As RIG-I has been shown to require a 5′-triphosphate group for sensitive detection of RNA transcripts were dephosphorylated prior to transfection and resulted in an ∼30% reduction in IFN-β mRNA levels at 8 h compared to mock treated RNA (Figure 2E)

**Figure 3.**
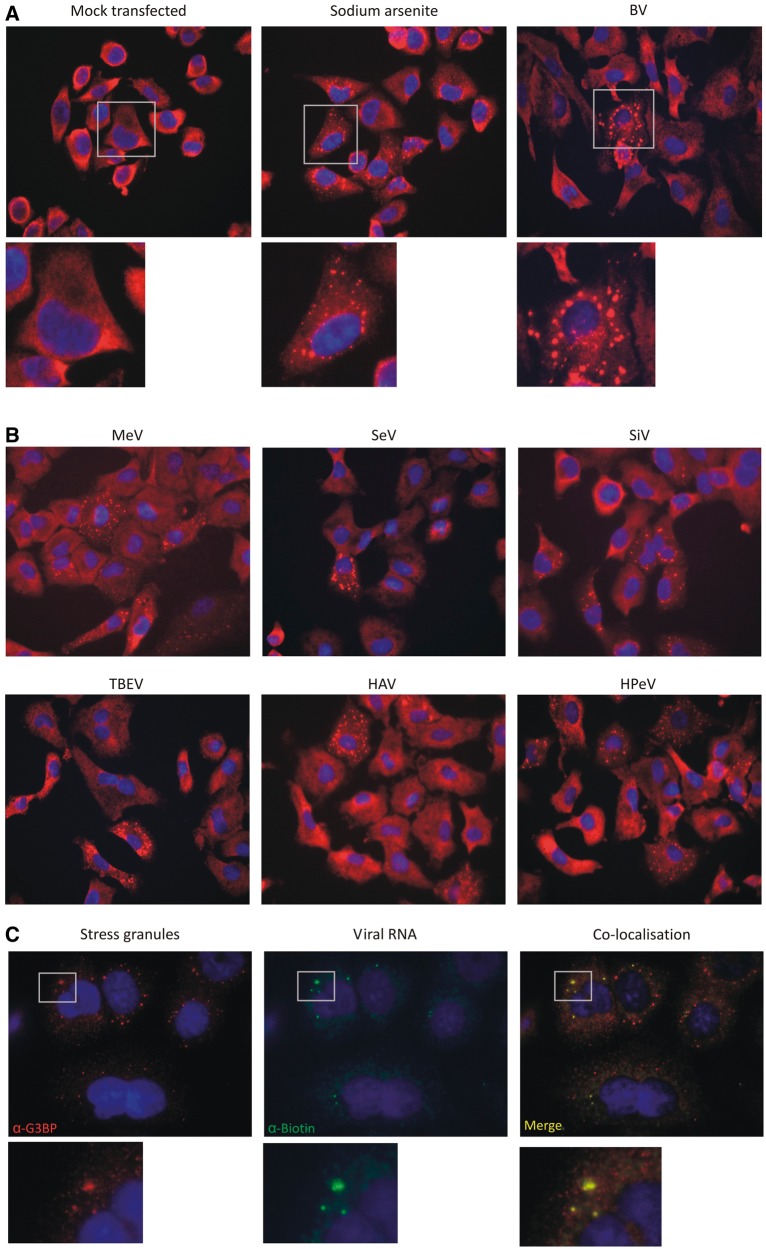
SG induction by transfected viral RNA. (**A**) SGs identified by immunodetection of G3BP (red; punctuate staining) following transfection with BV RNA and sodium arsenite treatment (positive control). (**B**) Reproducible induction of SGs by a panel of different viral RNA transcripts (**C**) Co-localization of SGs immunostained for G3BP (punctuate red dots) and for transfected viral RNA sequences containing biotin-labelled UTP followed by detection with an anti-biotin-CF633 conjugate (green). Inserts show precise co-localization of SGs and viral RNA (yellow).

Finally, evidence was obtained for a major role of PKR in the viral RNA recognition pathway; 80–85% reductions in IFN-β mRNA induction were observed in cells treated with 2-AP and the imidazolo-oxindole inhibitor, C16 (43,44) (Figure 2F). The involvement of PKR was further indicated by a substantial reduction in IFN-β induction after transfection of RNA into A549 cells expressing a PKR-specific shRNA compared to the parental cell line (Figure 2C). A role for PKR in the response to viral RNA is further supported by the observation of antiviral SG formation after transfection (Figure 3). PKR activation led to eIF2α phosphorylation and formation of SGs through recruitment of constitutively expressed cellular proteins such as G3BP (45). As a positive control, cells were treated with sodium arsenite, an inducer of oxidative stress which led to the appearance of large numbers of SGs identified through immunostaining for G3BP. This punctate appearance contrasted with that of mock cells which showed a diffuse cytoplasmic distribution of this marker (Figure 3A). Transfection of BV RNA induced a striking level of punctate G3BP-staining characteristic of SG formation. Similar induction of SGs was also observed by transfection of HPeV, HAV, MeV, SeV, SiV and TBEV RNA sequences (Figure 3B). Through transfection of biotin-labelled RNA, viral RNA co-localized within a proportion of SGs (Figure 3C).

**Figure 4.**
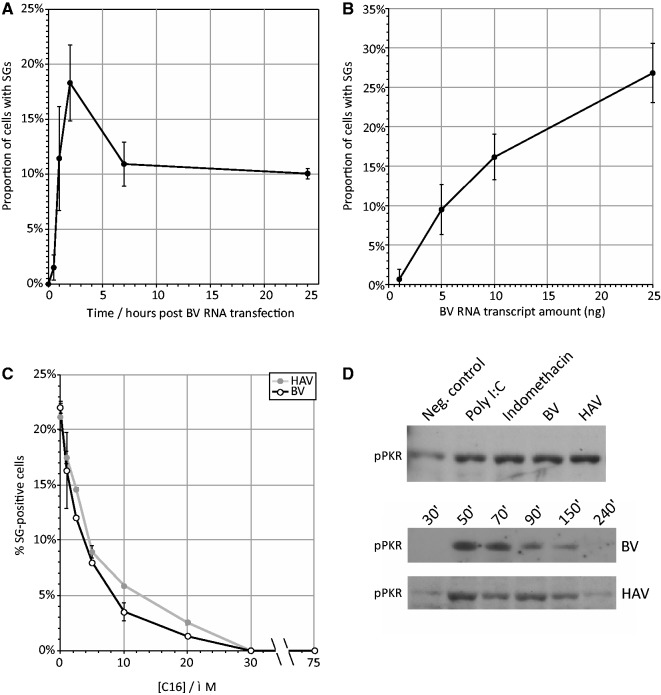
Time course and dose-dependence of SG induction. Frequency of SG-positive cells after transfection of viral BV RNA at (**A**) different time points post-transfection (**B**) differing transfected amounts of RNA and (**C**) susceptibility of SG induction by BV and HAV transcripts to the PKR inhibitor C16. All data points present the mean of three biological replicate with error bars showing SDs of replicate values. (**D**) Immunological detection of phosphorylated PKR (pPKR), showing BV and HAV transfected RNAs induce PKR phosphorylation at 150 min post transfection (top panel). Indomethacin is an inducer of PKR phosphorylation. Expression pattern following BV and HAV RNA transfection reveals the rapid onset and rise of pPKR expression (lower panel).

SG induction occurred independently of cellular IFN-β induction, since their formation was equivalent in A549 cells co-expressing N^Pro^ that inhibits IRF3 signalling (Supplementary Figure S1). SG formation occurred rapidly post-transfection (within 30 min and was maximal at 2 h; Figure 4A), substantially faster than the induction of IFN-β (Figure 1C). The frequencies of SGs induced by BV transcripts were dependent on RNA amounts transfected (Figure 4B). SG induction similarly showed a dose-dependent response to C16, a PKR phosphorylation inhibitor (Figure 4C) implying a direct or indirect role of PKR phosphorylation in the SG response to viral RNA sequences. Activation of PKR was detected in RNA-transfected cells by western blotting using phosphorylated-PKR specific antibodies (Figure 4D). Time-course experiments showed rapid induction of PKR phosphorylation after transfection of both BV and HAV transcripts (within 1 h), consistent with time course for the appearance of SGs. However, levels of phosphorylated PKR rapidly declined subsequently to background levels after 4 h (Figure 4D).

### Influence of RNA structure on viral RNA recognition

The previously described and characterized early cellular responses to viral RNA transfection were designed to model the immediate post-entry, pre-replication stages of virus infection on antiviral defences. To investigate whether RNA recognition was modulated by the presence of large scale RNA secondary structure, we generated a total of 18 different transcripts derived from RNA viruses with and without GORS, including two plant viruses with similarly structured genomes (Tables 1 and 2). These sequences covered a range of MFEDs (the percentage difference in folding energy between the native sequence and sequence order randomized controls) between 16.0% (CaCV) and −3.6% (BV), with a threshold of 3% dividing those into GORS (>3%) and non-GORS sequences (23,24).

**Figure 5.**
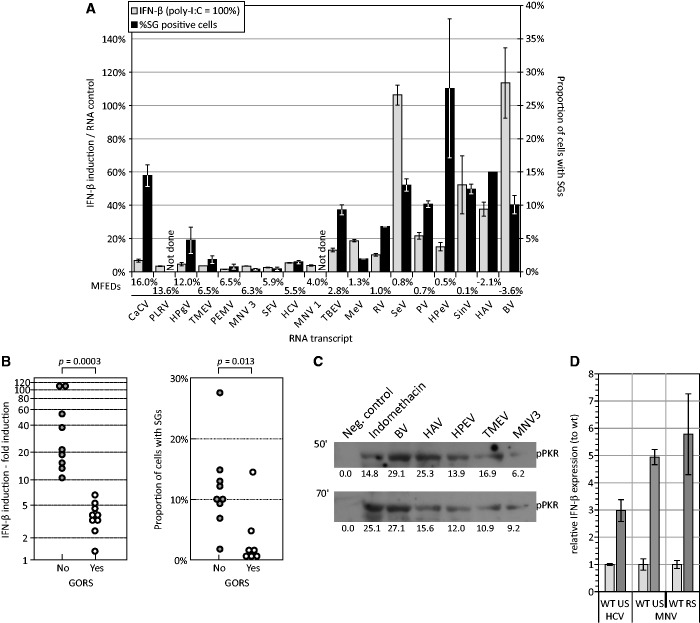
Relationship between IFN-β/ SG induction and RNA structure. (**A**) Induction of IFN-β (as a proportion of that induced by poly-I:C) and SG (frequencies of G3BP-positive cells) for transcripts from different viral genomes ranked from high to low by MFED value (*x*-axis). GORS threshold is considered to be 3% MFED. Bar heights represent the mean of at least two biological replicates; error bars show standard deviations. (**B**) Segregation of IFN-β and SG induction values by possession of GORS using 3% MFED threshold. *P-*values above graph show significance values calculated by Kruskall–Wallace non-parametric test. (**C**) Detection of phosphorylated PKR by western blot in cell lysates collected 50 min post-transfection of unstructured (BV, HAV and HPeV) and structured (TMEV, MNV3) transcripts. Numbers below bars indicate densitometry quantitation of target band (**D**) IFN-β response relative to that of wild type HCV and MNV transcripts with permuted sequences and of identical composition, coding and codon structure (US, RS). RNA secondary structure was disrupted in US transcripts with MFEDs of approximately zero; the RS transcript contained a (re-stabilized) synthetic RNA secondary structure with an MFED equal to that of WT virus Bar heights represent the mean of two biological replicates; error bars show SDs.

Transfection of this panel of viral RNAs showed that IFN-β mRNA induction was consistently greater using RNA transcripts with low MFED values (Figure 5A and B); all unstructured RNA generated fold inductions of >10 while all GORS RNAs induced >7 (*p* = 0.0003 by Kruskall–Wallace non-parametric test). In addition to RNA secondary structure, RNA transcripts additionally varied in their base composition, such as frequencies of mono- and di-nucleotides, frequencies of base pairs and ratios (Supplementary Table S3). Weaker associations with IFN-β response were observed for MFE, the frequency of A residues, CpG dinucleotides and G + C content) although these variables also showed compounding associations with each other and with MFED values. To investigate which of these five variables independently influenced IFN-β responses, IFN-β induction values were log transformed to approximate them to a normal distribution and multiple-linear regression was carried out (Table 3). Only MFED values were significantly independently associated with IFN-β induction once compounding effects were removed; differences in RNA folding accounted for 66% of the variability in IFN-β induction values (*R*^2^). A weaker association was observed between the presence of GORS and the frequency of SG induction (Figure 5A and B) although MFED values and SG frequencies did not display a significant relationship by single- or multiple-linear regression on analysis with the same compositional variables (data not shown). Although not as marked as the differences in IFN-β induction, cells transfected with unstructured BV, HAV and HPeV RNA sequences induced more phosphorylated PKR compared to structured RNAs (TMEV and MNV-3) (Figure 5D).

**Table 3.** RNA structure and composition varaiables influencing IFN-β response

*R* = 0.813			
Variable^a^	Co-efficient	*t*-statistic	*P*^b^
MFE	−0.00995	−0.47064	0.64
MFED	−8.16175	−2.57518	0.026*
Frequency A	2.088056	0.30285	0.77
G + C content	−5.27422	−0.91224	0.38
CpG	1.166543	0.97871	0.35
UpA	−0.60907	−0.63744	0.54

^a^List of variables showing individual associations with IFN-β induction.

^b^Two-tailed *P-*value.

To further demonstrate whether the observed differences in induction of IFN-β by RNA sequences were the result of differences in RNA secondary structure and not other compositional variables that might exist between them, synthetic HCV and MNV sequences were generated in which the degree of RNA secondary was adjusted while maintaining identical sequence compositions. Wild type (WT) HCV and MNV sequences of length 1.1–1.3 Kb derived from coding regions of their viral genomes were permuted in base order using an algorithm (CDLR) that retained mono- and di-nucleotide compositions along with coding and codon usage. Mutant sequences (US) with MFED values of −0.4% (MNV) and −1.3% (HCV) induced substantially greater IFN-β responses (3–5 fold) than the native sequences (MFED values of 9.0% and 10.9%, respectively) of transfection (Figure 5D). Intriguingly, a mutant MNV sequence [re-stabilized (RS)] selected from the upper part of the MFED distribution with an MFED value equal to that of the native sequence also showed substantial IFN-β induction. It appears that synthetic RNA secondary structure in the RS transcript did not modulate RNA recognition to the extent observed in native MNV sequence.

One explanation for the different cellular response to structured and unstructured RNA sequences is that they differ in stability once transfected into the cytoplasm. To investigate this, RNA levels were compared by quantitative PCR at 4 and 24 h post-transfection (Supplementary Figure S2). Both structured (HCV, TMEV and HPgV) and unstructured (BV) transcripts showed similar 2–4-fold declines in RNA levels between the two time points, thus providing no evidence for differences in their intra-cellular stability that would account for their different induction of IFN-β and SG formation.

**Figure 6.**
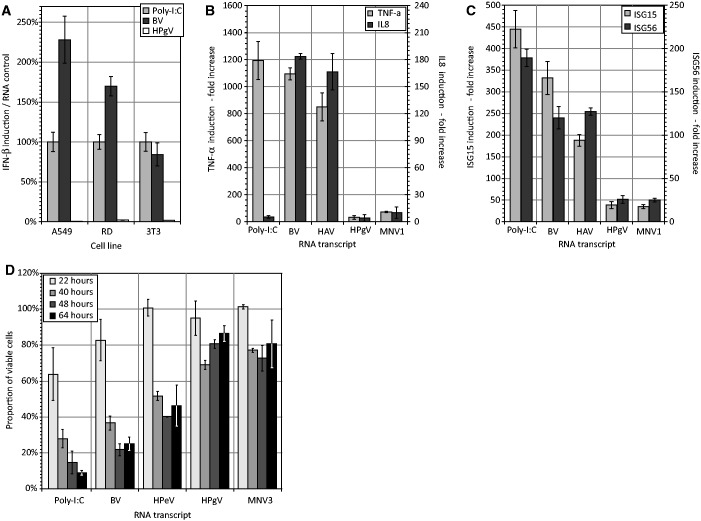
Differential induction of IFN-β and other cellular responses. (**A**) Differential induction of IFN-β in human (A549, RD) and murine (NIH3T3) cells. (**B**) Induction of TNF-α by unstructured (BV and HAV) and structured (HPgV and MNV1) RNA sequences. (**C**) Induction of the ISGs 15 and 56 by unstructured (BV and HAV) and structured (HPgV and MNV1) RNA sequences. (**D**) Effect of transfection of unstructured (BV and HPeV) and structured (HPgV and MNV3) RNA sequences on cell survival as assessed by viability assay. For all experiments, bar heights represent the mean of three biological replicates; error bars show SDs.

Similar differential induction of IFN-β mRNA by structured and unstructured RNA transcripts was observed in other cell types, including RD cells (human fibroblasts) and mouse 3T3 cells using transcripts from GORS (HPgV) and non-GORS (BV) viruses (Figure 6A). The differential induction of IFN-β induction and SG formation by structured and unstructured transcripts was also observed in other indices of cellular activation. Induction of TNF-α, IL8 and ISGs 15 and 56 were substantially greater in cells transfected with BV and HAV transcripts than by sequences from viruses with GORS, HPgV and MNV (Figure 6B and C). Likely related to the pro-apoptotic effect of TNF-α and IL8, unstructured viral transcripts (BV, HPeV) reduced cell viability by 60–80% after 24 h while >70% cells transfected with equal amounts of structured RNA (HPgV and MNV3) remained viable for 24 h and longer using a sensitive cell viability assay (Figure 6D).

**Figure 7.**
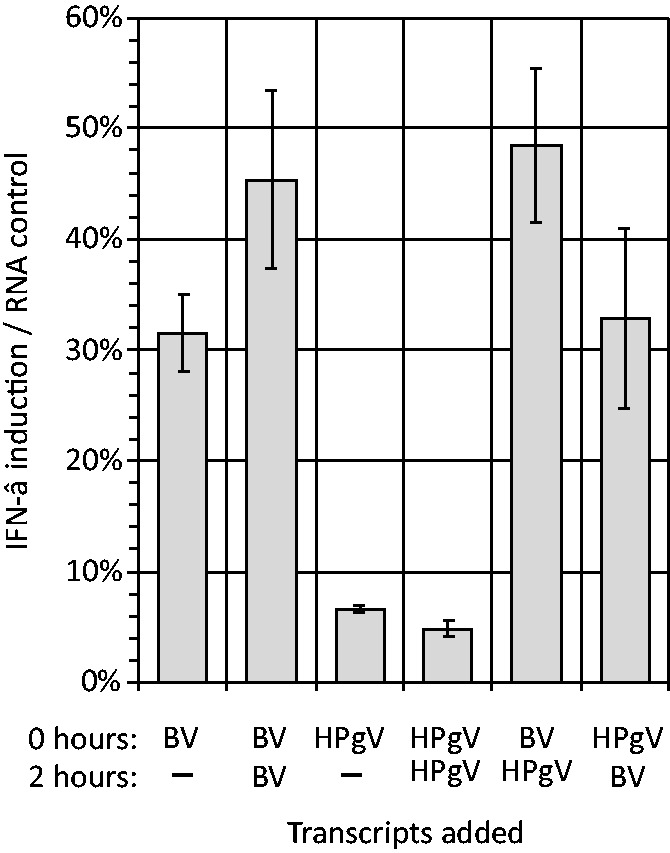
Intereference between structured- and unstructured-RNA transcripts on cellular IFN-β response. A549 fibroblasts were transfected with 9 ng of BV or HPgV RNA at time zero (line 1, *x*-axis), followed by repeat transfection at 2 h of 9 ng RNA (line 2). IFN-β induction (*y*-axis) was quantified relative to a poly-I:C control. Bar heights represent the mean of three biological replicates; error bars show SDs.

To investigate whether possession of genome-scale RNA structure inhibited recognition mechanisms or subsequent signalling pathways or alternatively, whether RNA folding prevented recognition by the cell, structured (HPgV) and unstructured (BV) transcripts were sequentially transfected into cells and IFN-β mRNA induction compared to that of transcripts transfected individually (Figure 7). IFN-β mRNA induction in response to single or sequential transfections of BV RNA was similar to that observed by transfecting HPgV RNA at 0 or 2 h by which time any inhibitory effect should have become apparent. The absence of *trans*-inhibition implies that GORS reduces detection by the cell rather than having a specific inhibitory function. Similar results were obtained when HPgV (structured) and BV (unstructured) RNA transcripts were co-transfected (data not shown).

## DISCUSSION

The association of GORS with virus persistence was originally described through observations that viruses with genomes possessing bioinformatically predicted extensive RNA secondary were those capable of establishing persist infections in their natural (immunocompetent) hosts (23,24). We have subsequently shown that experimental disruption of RNA secondary structure did not influence the replication ability of MNV in cell culture but profoundly reduced its fitness relative to WT virus on mouse infection (37). The lack of a cell culture phenotype for GORS-disrupted mutants, even in primary macrophage culture, may have originated at least in part from the ability of MNV to effectively evade or inhibit innate cell defences on infection. In common with other RNA (and DNA) viruses (2,3,46), MNV infection in cell culture induced minimal levels of IFN-β, ISG-15, -54, -56 and TNF-α (37). Attributes conferred by RNA secondary structure that contribute to evasion of innate cellular or inflammatory responses *in vivo* may therefore be partially or entirely redundant in a cell culture infection model.

Numerous studies report an absence of IFN induction by MNV, human enteroviruses and negative stranded RNA viruses such as influenza A virus and paramyxoviruses (34,37,47–49). For example, plaque-purified parainfluenza virus type 5 (PIV-5) lacking defective interfering particles remained able to infect A549 cells without inducing an IFN response irrespective of whether its V protein (that prevents MDA-5 recognition of dsRNA) was expressed or not (34). The conservation of V protein in PIV-5 isolates and the existence of homologues in other paramyxoviruses argue for an important role in its *in vivo* replication cycle, but it is one that exerts a minimal phenotype in existing cell culture models. Furthermore, the existence of multiple mechanisms—by an individual virus—for subverting innate responses creates the specific problem of dissecting the phenotypic effects of other contributors to control of cell defences by a wide range of viruses when studied *in vitro*. Besides recognition, there is some but not entirely consistent evidence that highly structured genomes might enable the virus to escape siRNA or miRNA coupled control mechanisms from the host cells (50–52).

Investigating the mechanisms underlying the undetectable effect of GORS disruption in MNV on its replication in cell culture despite its marked fitness attenuation *in vivo* formed the basis of the current study. To achieve this, we established a replication- and translation-free system in which interactions of viral RNA sequences with host defences could be directly investigated in the absence of active viral evasion strategies. The primary observation in the current study was that cellular responses to transcripts derived from naturally persistent viruses with structured RNA genomes were systematically different from those of RNA viruses with predominantly unstructured genomes (Figure 5). We further showed different responses to RNA transcripts permuted in base order and with differing degrees of RNA secondary structure but identical compositions. These findings imply that the range of cellular responses (IFN, SG) induced by transcripts from different viruses is not due to their composition features such as mono- and di-nucleotide frequencies, codon order or composition. Interpreting the physiological significance of this association and its contribution to virus host interactions is dependent on the nature of the cellular responses elicited by RNA transfection and their downstream effects on cellular defence and host-immune responses. A focus of the study was to therefore better understand RNA recognition mechanisms of viral RNA by the cell and the downstream pathways that are activated and mediate a cellular response.

Using a variety of different cell lines with defined defects in interferon pathway mediators and through the use of specific inhibitors, we were able to identify which steps in innate cellular defences responded to RNA transfection and which were differentially activated by structured and unstructured RNA. Although both single- and double-stranded RNA are substrates for binding to several different TLRs, the absence of expression of TLR7 and 8 on human fibroblasts (53) and the unaltered IFN induction in cells treated with chloroquine (Figure 2B) provided evidence against any of the TLRs being mediators of RNA transcript recognition in the current experimental system (41,54,55). Furthermore, we observed a complete dependence of IFN-β expression on IRF3 signalling, as demonstrated by the absence of its expression in cells expressing N^Pro^ which inhibits this pathway (Figure 2A). This rules out any significant role for TLR7 and 9 whose activation signals through the MyD88 / NF-κB pathway (53). These findings in fibroblasts do not, however, rule out their role in plasmacytoid dendritic cells and other antigen presenting cell types that participate in an augmented response to virus infections *in vivo*.

Recognition of transfected RNA by MDA-5 was similarly counter-indicated by observation of unaltered or actually increased IFN induction in cells where MDA-5 expression or function was inhibited (Figure 2A and C). These findings are consistent with the consensus view that MDA-5 primarily recognizes long dsRNA sequences typically associated with viral replication complexes, as well as poly-I:C (56,57). This PAMP is absent from the synthetic ssRNA transcripts generated in the current study (Figure 1D). Several studies however, report the ability of RIG-I to recognize ssRNA sequences although there is some ongoing uncertainty about the nature of the ligands required [reviewed in (58,59)]. In particular, it has been argued that some of the early evidence for RIG-I mediated recognition of single-stranded RNA arose through mis-primed, snap-back or hairpin transcripts of DNA templates by T7 polymerase (59). In our study, the close similarity of IFN-β responses to RNA before and after stringent denaturing gel purification (Figure 1D) provided evidence that IFN responses were not artefactually induced in this manner.

As discussed, PAMPs recognized by RIG-I include long dsRNA sequences and, at least in short, synthetic templates, ssRNA with a blunt ended double-stranded region adjacent to a triphosphorylated 5′ end (5′PPP) (60,61). The latter is compatible with recent structural models of RIG-I, in which sites of contact between dsRNA and a binding pocket for 5′PPP in the C-terminal regulatory domain have been identified (62–64). However, because the lengths of RNA that can by chemically synthesized are highly restricted, these *in vitro* experiments do not demonstrate whether the recognition of longer RNA sequences, such as viral genomes, is bounded by these stringent requirements. While the activation of RIG-I by several negative-stranded viruses appeared dependent of the exposure of the 5′PPP group and formation of a 5′/3′ pan-handle duplex sequence (60,65), other recent studies provide evidence for alternative recognition motifs. For example, purified genomic RNA from human immunodeficiency virus type 1 (HIV-1) was recognized by RIG-I even though it lacked 5′PPP and base-pairing at the 5′-end of the genomic RNA (66,67). Using a variety of RNA transcripts derived from the 5′- and 3′-ends of influenza A segment 6, IFN-β was potently induced through RIG-I activation by a range of predominantly single-stranded or partly double-stranded RNA sequences lacking pan-handle duplexes and which in many cases was independent of 5′ triphosphorylation (68). In another study, recognition of HCV genomic RNA with a 5′PPP end but which lacked a terminal 5′/3′ dsRNA sequence, was influenced by the presence of internal sequences such as the poly-U tract in the 3′UTR (69,70).

Consistent with the current study, a series of 800 base ssRNA transcripts spanning-coding regions of the HCV genome (that included the 4 kb regions analysed here) largely failed to induce IFN-β while the unstructured region in the 3′UTR (polyU/UC) induced IFN-β to levels greater than the poly-I:C control (69). In a similar study, ssRNA transcripts from several regions of the dengue virus genome acted as RIG-I-dependent PAMPs, although these were shielded from recognition when expressed as full length genome sequences (71). Although the authors equated regions of RNA folding with RIG-I activation, the genome of dengue is essentially unstructured in either sense or antisense orientation (mean MFED values for types 1–4 of 1.6% and 0.6%, respectively) and indeed most fragments appeared to reproduce the phenotype of unstructured RNAs in the current study (Figure 6). The evidence that we have obtained in the current study for a role of RIG-I in the recognition of the 4-Kb transcripts (Figure 2A, C and D) and the incomplete elimination of IFN induction using dephosphorylated transcripts (Figure 1E) are observations compatible with the evidence for more catholic RNA recognition mechanisms provided by these more recent studies.

Recognition of the 4-Kb transcripts was reduced but not eliminated in the RIG-I defective Huh7.5 cells (Figure 2D). These observations provide evidence for potential alternative, non-RIG-I, non-MDA5 and non-TLR3-coupled recognition methods that signalled through IRF3. A potential candidate PRR is PKR that is increasingly recognized as playing a major role in early virus recognition and activation of IFN-β responses (29). IFN induction was indeed dependent on PKR and largely abrogated through addition of PKR inhibitors 2AP and C16 (Figure 2E). The early involvement of PKR in RNA recognition was further revealed by differences in PKR phosphorylation by structured and unstructured RNA transcripts within 50 min after transfection and the differential induction of SG formation over the same time-scale. Their rapid appearance (Figure 5C) compared to IFN-β (Figure 1C) and their expression in cells with blocked IRF3 signalling (Supplementary Figure S1) rules out putative roles of IFN-β and interferon-stimulated gene (ISG) expression in their induction, consistent with previous observations (72). Indeed, the association between frequencies of SGs within cells with the degree of RNA secondary folding in the RNAs transfected (Figure 5A and B), implies a direct role of PKR-associated intrinsic responses in the differential response to transfected viral RNAs.

PKR is constitutively expressed in most cell types including fibroblasts (73,74) and upregulated by IFN signalling. It was initially ascribed a role in the host switch-off of cap-dependent translation in virus-infected cells, mediated through binding of viral RNA replication complexes to the PKR dsRNA-recognition domain that led to phosphorylation and inactivation of the translation initiation factor, eIF2α (75–77). In this role, it was considered primarily as an IFN-stimulated effector gene and part of the antiviral response to virus infections induced by IFN and other innate responses. However, more recent data points towards an important role of PKR in viral recognition and in early intrinsic response to infection and other stresses (31,32,78–80).

PKR is now known to be able to recognize a much wider range of substrates than dsRNA, including single-stranded RNA containing internal stem-loops (29,81,82). Although by definition viral genomes with GORS possess extensive internal base pairing, the observed induction of IFN-β by the RS mutant of MNV (with an MFED equal to that of the native sequence but derived from an underlying synthetic RNA structure) provides evidence that the effect of GORS on cellular responses may require specific structural features in the folded RNA over and above simple folding. What these are remains entirely unknown at present and will require future, more extensive bioinformatics and biophysical mapping to determine the functional patterns of internal base-pairing of RNA in structured viral genomes. We speculate that this systematic organization of RNA shape and pairing interactions may contribute to avoidance of RNA recognition by PKR and thus mediates the marked phenotypic differences between structured and unstructured RNAs observed in the current study. An alternative possibility is that RNA secondary structures in GORS transcripts mimic those associated with active inhibition of PKR in the adenovirus VA_I_, Epstein–Barr virus EBER and HIV–TAR transcripts through binding of duplex regions in stem-loops to the dsRNA recognition domain of PKR (83–85). Whether this is also the mechanism by which GORS RNA avoids activating PKR is unclear. Large-scale physical mapping to determine the specific pairings in GORS RNA sequences has not been performed to date although analysis of folding patterns predicted by MFOLD and other algorithms indicated that duplexes are highly restricted in length, with most >8 bp and never exceeding 11 (Simmonds *et al.*, manuscript in preparation). Predicted structures do not obviously resemble those in known inhibitors of PKR. The observation that co-transfection of structured (HPgV) RNA did not prevent the response to unstructured BV RNA (Figure 7) indeed provides some evidence against the PKR inhibitor hypothesis. However, the precise contribution of defined RNA structure to avoidance of recognition by PKR and other PRRs remains an area clearly warranting further investigation.

Whether SGs participate in the IFN response (79) or not (86), they clearly show different degrees of induction on transfection of structured and unstructured RNA (Figure 5A and B). However, the relative contributions of RIG-I and PKR/SG responses to transfection to the cellular antiviral response remain uncertain. Onomoto *et al.* (79) detected co-localization of RIG-I in SGs and proposed that their formation contributed to the ability of RIG-I to recognize foreign RNA sequences. In this model, the SGs were seen as macromolecular complexes involved in subsequent interactions with MAVS and downstream IFN-β induction. The idea of a recognition structure is attractive in further providing the milieu for the recently identified enhancement of dsRNA recognition through cleavage by RNAseL that generates further recognition motifs for RIG-I (87). On the other hand, while MDA-5 was recently also shown to co-localize in SGs induced by infection of cells by mouse cardio-viruses, inhibition of SG formation did not reduce its ability to signal recognition of viral dsRNA (86). Our observation that transfected viral RNA sequences co-localized in SGs (Figure 3C) and the involvement of both PKR and RIG-I in the IFN-β response is potentially more consistent with the Onomoto model. Evidence for the selective uptake of unstructured RNAs and their concentration in SGs provides evidence for active RNA recognition and channelling mechanisms of foreign RNA as part of the immediate intrinsic cellular response to infection.

In the evolutionary arms race between viruses and their hosts, the relatively simple, small, single-stranded, positive-sense RNA viruses have a potential Achilles heel in that the naked RNA genome is inevitably exposed—in the absence of any immunosuppressive viral proteins and before any are synthesized—early in the replication cycle. Those causing acute infections have evolved to outrun hosts defences. In contrast, we demonstrate here that some that cause persistent infections have evolved extensive RNA structure throughout the genomes which effectively prevents detection by cellular PRRs, in particular PKR. These studies contribute to a broader understanding of both sides of this arms race and further emphasizes the plethora of mechanisms that have evolved to detect, induce and evade innate immune responses.

## SUPPLEMENTARY DATA

Supplementary Data are available at NAR Online.

Supplementary Data
