# Supplementary material for: The influence of viral RNA secondary structure on interactions with innate host cell defences
Source: Nucleic Acids Res. 2013 Dec 13;42(5):3314–29. doi: 10.1093/nar/gkt1291 (PMC3950689; doi:10.1093/nar/gkt1291)
Supplement: Supplementary Data [file supp_42_5_3314__index.html]

The influence of viral RNA secondary structure on interactions with innate host cell defences — The influence of viral RNA secondary structure on interactions with innate host cell defences — Supplementary Data 

# The influence of viral RNA secondary structure on interactions with innate host cell defences

## Supplementary Data

files

**Files in this Data Supplement:**

- Supplementary Data - doc file
