# Supplementary material for: The influence of viral RNA secondary structure on interactions with innate host cell defences
Source: Nucleic Acids Res. 2013 Dec 13;42(5):3314–29. doi: 10.1093/nar/gkt1291 (PMC3950689; doi:10.1093/nar/gkt1291)
Supplement: Supplementary Data [file supp_gkt1291_nar-01783-a-2013-File009.doc]

SUPPLEMENTARY DATA TABLE S1

PRIMERS USED TO AMPLIFY DNA TEMPLATES FOR RNA TRANSCRIPTS

| **Virus** | **Primer** | **Sequence^1^** | **Length^2^** |
| --- | --- | --- | --- |
| CaCV | T7-CaCV S 144 | TAATACGACTCACTATAGGGGCAGTTTTCGAACTGGAAGTGTG | 4062 |
|  | CaCV AS 4205 | CAGGACTGTATTGGAGGGTAGC |  |
| PLRV | PLRV_S_4kb_T7 | TAATACGACTCACTATAGGGTCGATCCCAGGAATCACTTC | 4006 |
|  | PLRV_AS_4kb | CCAACATACTTCGCCCATCT |  |
| HPgV | T7-GBVC S 563 | TAATACGACTCACTATAGGGTCCTTCTGCTCCTTCTCGTG | 4011 |
|  | GBVC AS 4572 | GGGAGATGGTAATGGTGGGATCAAG |  |
| TMEV | T7-TMEV S 1163 | TAATACGACTCACTATAGGGATGGTTCCCTACGGACCTTC | 3999 |
|  | TMEV AS 5163 | TTTGTCCTAGATCATCCATAATCAC |  |
| PEMV-2 | T7-PEMV S 200 | TAATACGACTCACTATAGGGGGAGTGCAACCTCCTTATCC | 4023 |
|  | PEMV AS 4222 | AGGGAGGAAGAAGCACAACCGGTCC |  |
| MNV | T7-MNV S 147 | TAATACGACTCACTATAGGGTTACATGACCCCTCCTGAGC | 4014 |
|  | MNV AS 4090 | TTTATCTTCCCGTAGATCTTGTCTG |  |
| SFV | T7-SFV S 167 | TAATACGACTCACTATAGGGAGGTGGAGTCATTGCAGGTC | 4102 |
|  | SFV AS 4279 | ATGACTGTTTTAATTGTGCCCACTG |  |
| HCV | T7-HCV S 594 | TAATACGACTCACTATAGGGGGCCCCTATATGGGAATGAG | 4035 |
|  | HCV AS 4622 | AGACGTCCAACCCTCTATAGTATGC |  |
| TBEV | TBEV_S_4kb_T7 | TAATACGACTCACTATAGGGGCACTCGCAGTGGCCTCGTT | 4315 |
|  | TBEV_AS_4kb | ttTTGGTGCCGTGCGGTAGCTG |  |
| MeV | MeV_S_4kb_T7 | TAATACGACTCACTATAGGGCGGAAGAACAAGGCTCAGAC | 3995 |
|  | MeV_AS_4kb | GATTGATGGCTGGAACGAGT |  |
| SeV | SeV T7 S 2911 | TAATACGACTCACTATAGGACGTGCCCTAAAGTCTGCAA | 3925 |
|  | SeV AS 6834 | ACAAAGCCCACTGGGTGAAT |  |
| RV | T7-RV-1048(S) | TAATACGACTCACTATAGGGCATGCTGTCCTACTGCAAGA | 4067 |
|  | RV-AS-5081 | GATTCTTGTTGAGCAGGTGAC |  |
| PV | T7-PV S 850 | TAATACGACTCACTATAGGGTCAGCTAGTAACGCGGCTTC | 3941 |
|  | PV AS 4788 | GTGGATGCTAGAACGTAATTTGAAG |  |
| HPeV 1 | HPeV_S_4KB_T7 | TAATACGACTCACTATAGGGAAGCGTCGGTAATGAAATTGGAGGT | 4092 |
|  | HPeV_AS_4Kb | ggTGGTATGGCATCTGGGAAATTGTGC |  |
| SiV | SinV T7 S 2008 | TAATACGACTCACTATAGGGCCCCGCCAAGAATACAGAA | 3965 |
|  | SinV AS 5941 | TCGGTGGGCATCATCTGGTA |  |
| HAV | HAV_S_T7 | TAATACGACTCACTATAGGGcatgctcttttccatgaagttg | 4002 |
|  | HAV_AS | Ttatcatctgaaattccctgagac |  |
| BV | T7-BV S 165 | TAATACGACTCACTATAGGGTATTTTGGTCGGGAGCTTTG | 4093 |
|  | BV AS 4248 | TACTTCGCATATCACTTGTTTCTCC |  |
| HAV-1kb | HAV-AS-2001 | gaacacgaaatctcaaRgttgactg | 951 |
| HAV-2kb | HAV-AS-3215 | catttgacaaYtcttcctgagcata | 2165 |
| HAV-3kb | HAV-AS-4081 | gcatccttRaaacttttaaaaat | 3031 |
| HPeV 1-1kb | HPEV-AS-1823 | CCATCACTGAGAATAACTGGGCT | 1024 |
| HPeV 1-2kb | HPEV-AS-2805 | ATCTGCTCTCCGGCTGGTA | 2006 |
| HPeV 1-3kb | HPEV-AS-3759 | TCTGCCACAATTTCAGCCAGT | 2960 |

^1^ T7 site underlined

^2^ Amplicon length

SUPPLEMENTARY DATA TABLE S2

PRIMERS USED TO QUANTIFY GENE EXPRESSION

| **Gene** | **Forward** | **Reverse** | **Reference** |
| --- | --- | --- | --- |
| **Human** |  |  |  |
| GAPDH | GAAATCCCATCACCATCTTCCAGG | GAGCCCCAGCCTTCTCCATG | 1 |
| IFIT2 | CGAACAGCTGAGAATTGCAC | CAAGTTCCAGGTGAAATGGC | 1 |
| IFN-β | GAACTTTGACATCCCTGAGGAG | GCCAGGAGGTTCTCAACAATAG | 1 |
| TNF-α | GGAGAAGGGTGACCGACTCA | CTGCCCAGACTCGGCAA | 1 |
| ISG15 | GGCGGGCAACGAATTCCAGGTGT | CTCCCCGCAGGCGCAGATTCA | 1 |
| ISG56 | CTTGAGCCTCCTTGGGTTCG | GCTGATATCTGGGTGCCTAAGG | 1 |
| 18S | GTAACCCGTTGAACCCCATTC | ACCATCCAATCGGTAGTAGCG | 2 |
| **Mouse** |  |  |  |
| IFN-β | CACAGCCCTCTCCATCAACT | GCATCTTCTCCGTCATCTCC | 3 |

SUPPLEMENTARY DATA TABLE S3

COMPOSITIONAL AND RNA SECONDARY VARIABLES

USED FOR ANALYSIS OF IFN- β RESPONSES TO RNA TRANSCRIPTS

| **Virus** | ***Outcome*** | ***RNA Structure*** | | ***Mononucleotide frequencies*** | | | | ***Combinations of bases*** | | | | | | ***Ratios of bases*** | | | |
| --- | --- | --- | --- | --- | --- | --- | --- | --- | --- | --- | --- | --- | --- | --- | --- | --- | --- |
|  | **IFN-β** | **MFE** | **MFED** | **fA** | **fC** | **fG** | **fU** | **fA+C** | **fA+G** | **fA+U** | **fC+G** | **fC+U** | **fG+U** | **A/U** | **C/G** | **U/C** | **A/G** |
| CaCV | 6.76 | -77.0 | 16.0% | 0.279 | 0.252 | 0.216 | 0.254 | 0.531 | 0.495 | 0.533 | 0.468 | 0.506 | 0.47 | 1.098 | 1.167 | 0.992 | 1.292 |
| PLRV | 3.41 | -84.2 | 13.6% | 0.281 | 0.257 | 0.251 | 0.211 | 0.538 | 0.532 | 0.492 | 0.508 | 0.468 | 0.462 | 1.332 | 1.024 | 1.218 | 1.120 |
| HPgV | 4.66 | -115.1 | 12.0% | 0.167 | 0.266 | 0.328 | 0.239 | 0.433 | 0.495 | 0.406 | 0.594 | 0.505 | 0.567 | 0.699 | 0.811 | 1.113 | 0.509 |
| TMEV | 3.63 | -73.7 | 6.5% | 0.234 | 0.289 | 0.213 | 0.265 | 0.523 | 0.447 | 0.499 | 0.502 | 0.554 | 0.478 | 0.883 | 1.357 | 1.091 | 1.099 |
| PEMV | 1.54 | -93.7 | 6.5% | 0.225 | 0.278 | 0.281 | 0.216 | 0.503 | 0.506 | 0.441 | 0.559 | 0.494 | 0.497 | 1.042 | 0.989 | 1.287 | 0.801 |
| MNV3 | 3.41 | -99.1 | 6.3% | 0.214 | 0.288 | 0.291 | 0.207 | 0.502 | 0.505 | 0.421 | 0.579 | 0.495 | 0.498 | 1.034 | 0.990 | 1.391 | 0.735 |
| SFV | 2.59 | -84.4 | 5.9% | 0.273 | 0.25 | 0.276 | 0.201 | 0.523 | 0.549 | 0.474 | 0.526 | 0.451 | 0.477 | 1.358 | 0.906 | 1.244 | 0.989 |
| HCV | 5.35 | -101.9 | 5.5% | 0.188 | 0.297 | 0.291 | 0.224 | 0.485 | 0.479 | 0.412 | 0.588 | 0.521 | 0.515 | 0.839 | 1.021 | 1.326 | 0.646 |
| MNV1 | 3.80 | -96.2 | 4.0% | 0.214 | 0.281 | 0.291 | 0.214 | 0.495 | 0.505 | 0.428 | 0.572 | 0.495 | 0.505 | 1.000 | 0.966 | 1.313 | 0.735 |
| TBE | 13.05 | -93.8 | 2.8% | 0.235 | 0.224 | 0.325 | 0.216 | 0.459 | 0.56 | 0.451 | 0.549 | 0.44 | 0.541 | 1.088 | 0.689 | 1.037 | 0.723 |
| MV | 18.71 | -70.2 | 1.3% | 0.293 | 0.299 | 0.235 | 0.174 | 0.592 | 0.528 | 0.467 | 0.534 | 0.473 | 0.409 | 1.684 | 1.272 | 1.718 | 1.247 |
| RV | 10.23 | -118.2 | 1.0% | 0.143 | 0.397 | 0.319 | 0.141 | 0.54 | 0.462 | 0.284 | 0.716 | 0.538 | 0.46 | 1.014 | 1.245 | 2.816 | 0.448 |
| SeV | 106.23 | -72.0 | 0.8% | 0.293 | 0.227 | 0.243 | 0.237 | 0.52 | 0.536 | 0.53 | 0.47 | 0.464 | 0.48 | 1.236 | 0.934 | 0.958 | 1.206 |
| PV | 21.61 | -70.3 | 0.7% | 0.293 | 0.246 | 0.229 | 0.232 | 0.539 | 0.522 | 0.525 | 0.475 | 0.478 | 0.461 | 1.263 | 1.074 | 1.060 | 1.279 |
| HPeV | 15.12 | -62.2 | 0.5% | 0.318 | 0.192 | 0.2 | 0.29 | 0.51 | 0.518 | 0.608 | 0.392 | 0.482 | 0.49 | 1.097 | 0.960 | 0.662 | 1.590 |
| SiV | 52.28 | -71.6 | 0.1% | 0.286 | 0.261 | 0.252 | 0.202 | 0.547 | 0.538 | 0.488 | 0.513 | 0.463 | 0.454 | 1.416 | 1.036 | 1.292 | 1.135 |
| HAV | 37.63 | -61.7 | -2.1% | 0.311 | 0.156 | 0.21 | 0.322 | 0.467 | 0.521 | 0.633 | 0.366 | 0.478 | 0.532 | 0.966 | 0.743 | 0.484 | 1.481 |
| BV | 113.48 | -51.2 | -3.6% | 0.374 | 0.151 | 0.184 | 0.291 | 0.525 | 0.558 | 0.665 | 0.335 | 0.442 | 0.475 | 1.285 | 0.821 | 0.519 | 2.033 |

SUPPLEMENTARY DATA TABLE S3 (continued)

| **Virus** | ***Dinucleotide frequencies*** | | | | | | | | | | | | | | | |
| --- | --- | --- | --- | --- | --- | --- | --- | --- | --- | --- | --- | --- | --- | --- | --- | --- |
|  | **f_ApA** | **f_ApC** | **f_ApG** | **f_ApU** | **f_CpA** | **f_CpC** | **f_CpG** | **f_CpU** | **f_GpA** | **f_GpC** | **f_GpG** | **f_GpU** | **f_UpA** | **f_UpC** | **f_UpG** | **f_UpU** |
| CaCV | 0.095 | 0.072 | 0.045 | 0.067 | 0.081 | 0.065 | 0.042 | 0.065 | 0.068 | 0.049 | 0.047 | 0.052 | 0.036 | 0.067 | 0.082 | 0.069 |
| PLRV | 0.093 | 0.062 | 0.072 | 0.055 | 0.076 | 0.071 | 0.052 | 0.059 | 0.074 | 0.061 | 0.068 | 0.047 | 0.039 | 0.063 | 0.059 | 0.05 |
| HPgV | 0.029 | 0.05 | 0.047 | 0.04 | 0.056 | 0.083 | 0.06 | 0.067 | 0.059 | 0.074 | 0.122 | 0.073 | 0.023 | 0.059 | 0.099 | 0.059 |
| TMEV | 0.063 | 0.071 | 0.047 | 0.053 | 0.078 | 0.089 | 0.036 | 0.086 | 0.056 | 0.056 | 0.053 | 0.048 | 0.037 | 0.073 | 0.077 | 0.079 |
| PEMV | 0.055 | 0.063 | 0.066 | 0.041 | 0.07 | 0.09 | 0.058 | 0.06 | 0.063 | 0.068 | 0.085 | 0.065 | 0.037 | 0.057 | 0.073 | 0.049 |
| MNV3 | 0.047 | 0.053 | 0.067 | 0.048 | 0.071 | 0.097 | 0.051 | 0.069 | 0.076 | 0.082 | 0.089 | 0.044 | 0.02 | 0.056 | 0.085 | 0.046 |
| SFV | 0.073 | 0.074 | 0.079 | 0.047 | 0.076 | 0.062 | 0.059 | 0.053 | 0.079 | 0.069 | 0.067 | 0.062 | 0.045 | 0.045 | 0.072 | 0.04 |
| HCV | 0.029 | 0.063 | 0.05 | 0.046 | 0.074 | 0.089 | 0.059 | 0.075 | 0.052 | 0.08 | 0.1 | 0.06 | 0.034 | 0.065 | 0.082 | 0.043 |
| MNV1 | 0.046 | 0.051 | 0.065 | 0.051 | 0.069 | 0.091 | 0.049 | 0.072 | 0.076 | 0.081 | 0.091 | 0.043 | 0.023 | 0.059 | 0.085 | 0.048 |
| TBE | 0.054 | 0.059 | 0.074 | 0.048 | 0.073 | 0.051 | 0.038 | 0.062 | 0.089 | 0.064 | 0.111 | 0.062 | 0.019 | 0.05 | 0.102 | 0.045 |
| MV | 0.086 | 0.071 | 0.078 | 0.058 | 0.097 | 0.109 | 0.041 | 0.051 | 0.08 | 0.057 | 0.067 | 0.032 | 0.031 | 0.061 | 0.049 | 0.033 |
| RV | 0.02 | 0.06 | 0.041 | 0.022 | 0.059 | 0.137 | 0.143 | 0.058 | 0.051 | 0.15 | 0.078 | 0.041 | 0.014 | 0.051 | 0.057 | 0.019 |
| SeV | 0.075 | 0.063 | 0.081 | 0.074 | 0.081 | 0.054 | 0.029 | 0.064 | 0.082 | 0.044 | 0.065 | 0.052 | 0.055 | 0.066 | 0.069 | 0.047 |
| PV | 0.079 | 0.081 | 0.067 | 0.066 | 0.092 | 0.064 | 0.032 | 0.058 | 0.065 | 0.049 | 0.061 | 0.055 | 0.058 | 0.052 | 0.069 | 0.053 |
| HPeV | 0.103 | 0.061 | 0.066 | 0.087 | 0.084 | 0.044 | 0.009 | 0.056 | 0.068 | 0.035 | 0.047 | 0.051 | 0.063 | 0.053 | 0.079 | 0.096 |
| SiV | 0.083 | 0.072 | 0.08 | 0.051 | 0.08 | 0.076 | 0.057 | 0.049 | 0.078 | 0.065 | 0.057 | 0.053 | 0.045 | 0.049 | 0.058 | 0.049 |
| HAV | 0.101 | 0.047 | 0.07 | 0.093 | 0.074 | 0.028 | 0.002 | 0.053 | 0.08 | 0.027 | 0.05 | 0.054 | 0.057 | 0.055 | 0.089 | 0.123 |
| BV | 0.136 | 0.05 | 0.077 | 0.112 | 0.075 | 0.021 | 0.007 | 0.048 | 0.077 | 0.036 | 0.032 | 0.04 | 0.087 | 0.045 | 0.068 | 0.091 |

SUPPLEMENTARY DATA FIGURE S1


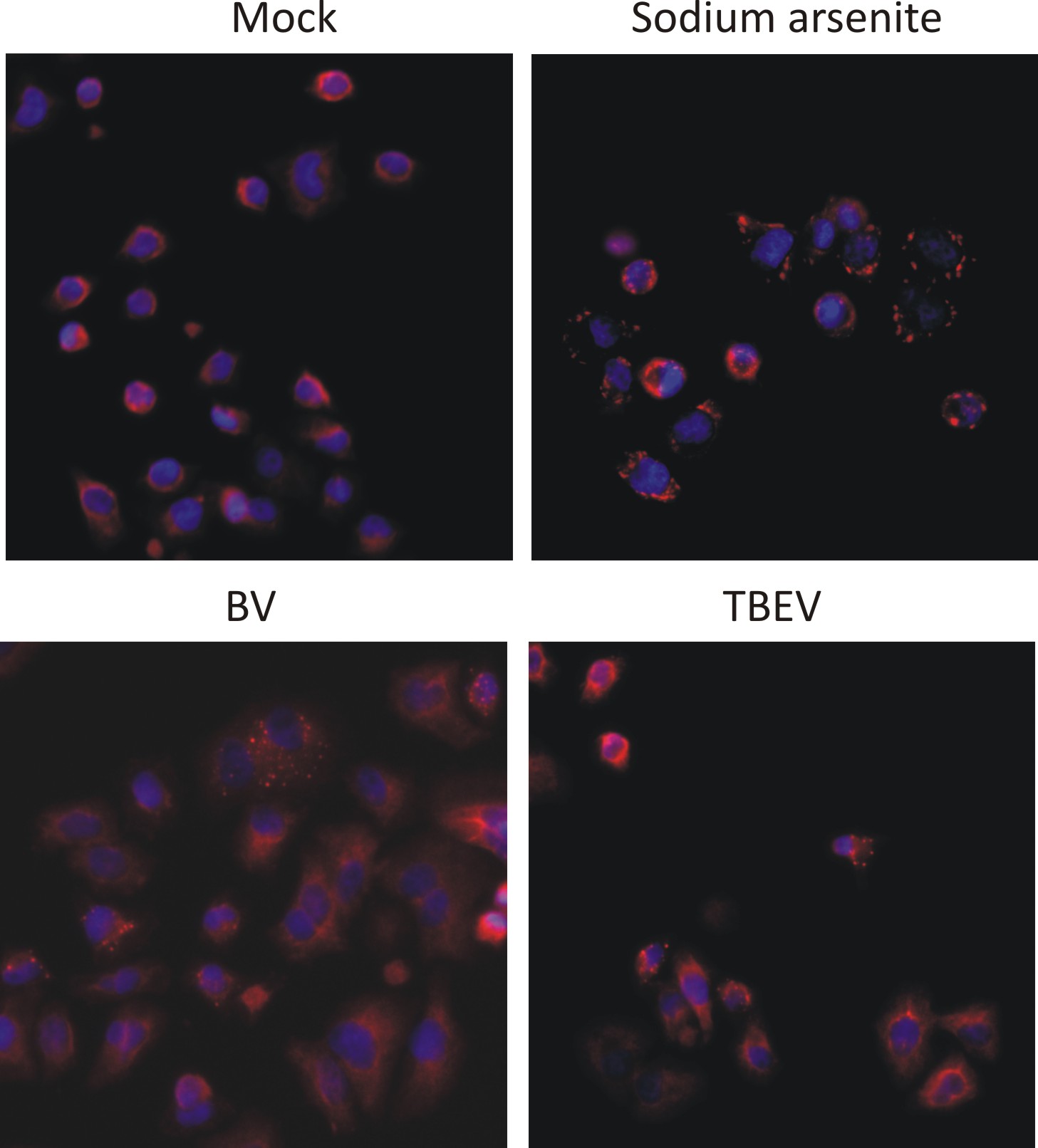


Continued induction of SGs in A549 cells transfected with viral RNA sequences with IRF3-mediated signalling inhibited by N^Pro^

SUPPLEMENTARY DATA FIGURE S2


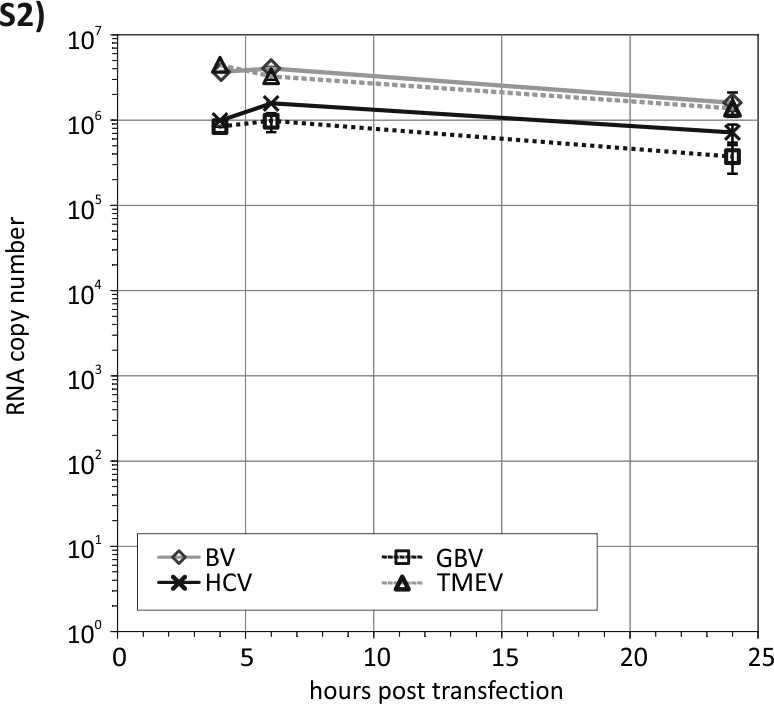


Stability of RNA transcripts after transfection into A549 cells. RNA levels in cell lysates were determined by transcript-specific qPCR assays for HCV, HPgV, TMEV and BV RNA sequences.
